# LC-HRMS/MS-Guided Profiling and Biological Evaluation of *Stachys duriaei* Extracts: Anticancer and Vasorelaxant Mechanisms via Apoptosis and Endothelium-Dependent Pathways

**DOI:** 10.3390/molecules30173570

**Published:** 2025-08-31

**Authors:** Racha Lydia Bouchouka, Zahia Kabouche, Marie Defondaumière, Marlène Debiossat, Catherine Ghezzi, Laurent Riou, Tarek H. Taha, Fehmi Boufahja, Hamdi Bendif, Stefania Garzoli

**Affiliations:** 1 Laboratoire d’Obtention de Substances Thérapeutiques, Université de Constantine 1, Frères Mentouri, Campus Chaabet Ersas, Constantine 25000, Algeria; rachabouchouka@gmail.com (R.L.B.); zahiakabouche@umc.edu.dz (Z.K.); 2 INSERM, LRB, Université de Grenoble Alpes, 38000 Grenoble, France; marie.caroline.defondaumiere@gmail.com (M.D.); marlene.debiossat@univ-grenoble-alpes.fr (M.D.); catherine.ghezzi@univ-grenoble-alpes.fr (C.G.); laurent.riou@univ-grenoble-alpes.fr (L.R.); 3 Department of Biology, College of Science, Imam Mohammad Ibn Saud Islamic University (IMSIU), Riyadh 11623, Saudi Arabia; thali@imamu.edu.sa (T.H.T.); faboufahja@imamu.edu.sa (F.B.); 4 Department of Chemistry and Technologies of Drug, Sapienza University, 00185 Rome, Italy

**Keywords:** *Stachys duriaei*, Antiproliferative, Apoptosis, LC-HRMS/MS, cell cycle, vascular reactivity

## Abstract

*Stachys duriaei* (Lamiaceae) remains unexplored despite its pharmacological potential. In this study, for the first time, the antiproliferative, pro-apoptotic, cell cycle arrest, and vasorelaxant effects of the *n*-butanolic extract (BESD) and a VLC fraction (BF1SD) of *Stachys duriaei* were investigated. Antiproliferative activity was evaluated on PC3 and MDA-MB-231 cell lines via MTT assay (72 h). Apoptosis (Annexin V-FITC/PI) and cell cycle arrest (PI/RNase) were assessed by flow cytometry (24 h, 250–1000 µg/mL). Vasorelaxant effects were studied ex vivo on rat aortic rings. LC-HRMS/MS was used for phytochemical analysis. BESD showed higher antiproliferative activity (IC_50_: 196 ± 6 µg/mL for PC3, 182 ± 8 µg/mL for MDA-MB-231) than BF1SD (IC_50_: 281 ± 6 µg/mL and 273 ± 3 µg/mL, respectively). Apoptosis was dose-dependent, with BF1SD displaying a stronger effect at 1000 µg/mL (67.3 ± 0.5% vs. 49.9 ± 0.7% for BESD). BESD induced G2/M arrest, while BF1SD caused G0/G1 and G2/M arrest. Vasorelaxation was endothelium-dependent, likely mediated by NO. Identified compounds (hyperoside, luteolin-7-glucoside, and rutin) may contribute to these effects. BESD and BF1SD exhibit anticancer and vasorelaxant properties, indicating potential therapeutic use against cancer and cardiovascular diseases. Further studies are needed to isolate active compounds and confirm their effects in vivo.

## 1. Introduction

Cardiovascular diseases and cancer are major causes of morbidity and mortality worldwide. According to the World Heart Report 2023, cardiovascular diseases caused 20.5 million deaths in 2021, accounting for nearly one-third of all global deaths [1]. According to estimates from the World Health Organization (WHO), cancer caused 9.7 million deaths worldwide in 2022 [2]. These figures highlight the urgent need to develop new therapeutic approaches that are both effective and well-tolerated. Natural products, particularly those derived from medicinal plants, represent a promising source of bioactive compounds with anticancer and cardioprotective properties. Among these plants, the *Stachys* genus (Lamiaceae), which includes more than 300 species primarily found in Mediterranean and temperate regions, has attracted growing interest due to its diverse pharmacological activities [3]. Recent studies have highlighted the antioxidant and anti-inflammatory properties of certain *Stachys* species. For example, the phytochemical analysis of *Stachys arvensis* revealed a high content of polyphenols, particularly flavonoids such as hyperoside and panasenoside, contributing to significant antioxidant activity [4]. Furthermore, studies conducted by Benmebarek et al. [5] have demonstrated the immunostimulatory effects of the *n*-butanolic extract of *Stachys mialhesi*, suggesting a therapeutic potential for the treatment of inflammatory diseases. Although the species *Stachys duriaei* has not been studied at all, research on other species of the genus *Stachys* has revealed a composition rich in secondary metabolites, particularly phenolic compounds and flavonoids. In continuation of works on *Stachys* species [6,7,8,9,10,11], this study aims to explore the biological effects of the *n*-butanolic extract (BESD) and the VLC fraction of the *n*-butanolic extract (BF1SD) of *S. duriaei*, with a particular focus on their antiproliferative activity, their ability to induce apoptosis, and their impact on vascular reactivity. Thus, the main objective of this work is to characterize the phytochemical composition of the BESD and the BF1SD through LC-HRMS/MS and to evaluate their biological effects on cancer cell lines and ex vivo vascular tissues. This approach will not only deepen the understanding of the mechanisms of action of these extracts but also open new perspectives for their potential use as natural therapeutic agents in the treatment of cancer and cardiovascular diseases.

## 2. Results

### 2.1. LC-HRMS/MS Analyses of the BESD and BF1SD

**Figure 1:**
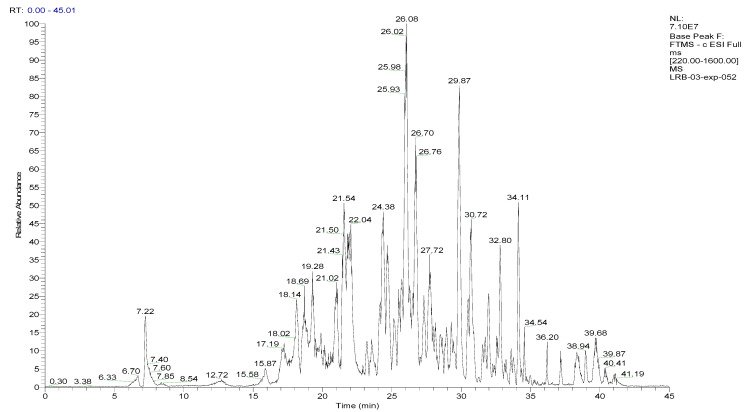
LC–ESI–DDA–HRMS base peak chromatogram of BESD in negative ion mode.

**Figure 2:**
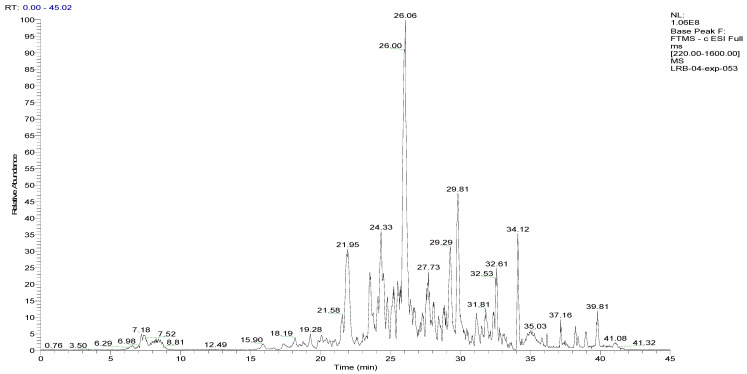
LC–ESI–DDA–HRMS base peak chromatogram of BF1SD in negative ion mode.

To analyze the phytochemical composition of the BESD and BF1SD, UHPLC-DAD-ESI/HRMS-MS analyses were performed. The results indicated the presence of 66 compounds in the BESD and 69 compounds in the BF1SD (Table 1 and Table S1, Figure 1 and Figure 2). The identification of metabolites was carried out using the GNPS platform. The results indicated that BESD and BF1SD extracts were rich in secondary metabolites, mainly belonging to flavonoids and phenolic acids.

### 2.2. Compounds Detected in the BESD

Thirteen flavonoids belonging to the flavone, flavanol, and glycosylated flavonoid families were detected in the BESD with hyperoside (11.41%), luteolin-7-*O*-β-D-glucoside (5.27%), peltatoside (quercetin-3-*O*-arabinoglucoside) (5.07%), 2’-*O*-galloylhyperin (5.14%), and kaempferol-3-*O*-rutinoside (2.08%) as the major components. Regarding phenolic acids, the predominant compounds were 1,6-bis-*O*-galloyl-β-D-glucose (5.04%) and 1,2,6-Trigalloylglucose (2.16%). Other bioactive metabolites belonging to various phytochemical classes further contributed to the composition of the BESD (Table 1).

### 2.3. Compounds Detected in the BF1SD

The BF1SD was characterized by the presence of twelve flavonoids belonging to the flavone, flavanol, and glycosylated flavonoid families, although their concentrations slightly differ from those in the BESD. The most abundant flavonoids were sayaendoside (6.62%), hyperoside (5.00%), rutin (4.58%), tetrahydroxy-flavone (3.98%), and peltatoside (2.19%). The highest concentrations of phenolic acids were found in Hydroxysafflor Yellow A (8.07%), [6-[2-(3,4-dihydroxyphenyl)-8-hydroxy-4-oxochromen-7-yl]oxy-3,4,5-trihydroxyoxan-2-yl]methyl(*E*)-3-(4-hydroxyphenyl)prop-2-enoate (4.70%), and 1,6-bis-*O*-galloyl-β-D-glucose (0.18%). Unlike the BESD, the BF1SD contained a significant amount of melibiose (2.94%), a disaccharide belonging to the bioactive carbohydrate family, which may play a role in the stabilization and absorption of other bioactive metabolites. The secondary metabolites identified in the BESD and BF1SD revealed a heterogeneous chemical composition, primarily composed of flavonoids, bioactive carbohydrates, and phenolic acids. The potential interaction between these different compound classes could confer promising biological properties to these extracts (Table 1).

### 2.4. Antiproliferative Effect of the BESD and BF1SD on PC3 and MDA-MB-231 Cell Lines

**Figure 3:**
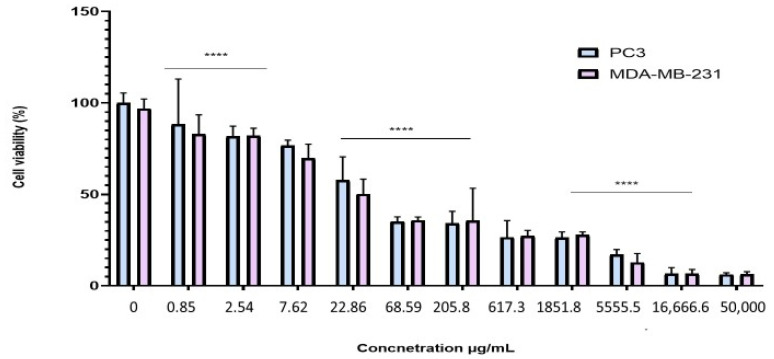
Dose-dependent effect of BESD on PC3 and MDA-MB-231 cancer cells. Cell viability was determined using an MTT assay and expressed as a percentage. Cells were treated with BESD at different concentrations for 72 h. Data are presented as mean ± standard deviation (*n* = 3), **** *p* < 0.0001. Statistical analysis was performed using two-way ANOVA followed by Bonferroni’s correction. BESD: *n*−butanolic extract of *Stachys duriaei*.

**Figure 4:**
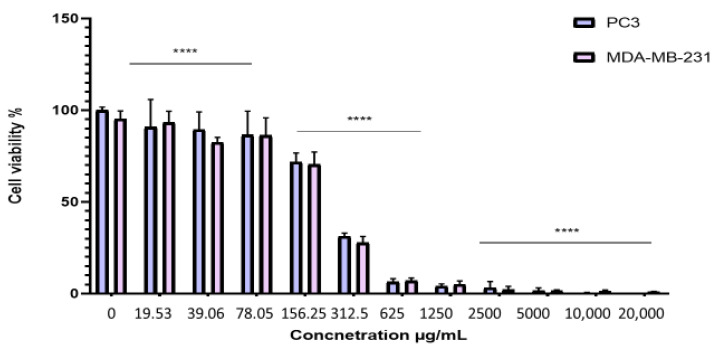
Dose-dependent effect of BF1SD on PC3 and MDA-MB-231 cancer cells. Cell viability was determined using an MTT assay and expressed as a percentage. Cells were treated with BF1SD at different concentrations for 72 h. Data are presented as mean ± standard deviation (*n* = 3), **** *p* < 0.0001. Statistical analysis was performed using two-way ANOVA followed by Bonferroni’s correction. BF1SD: First fraction of the *n*−butanolic extract of *Stachys duriaei.*

To evaluate the antiproliferative effect of the BESD and BF1SD on prostate cancer (PC3) and breast cancer (MDA-MB-231) cell lines, the cells were treated with varying concentrations of the extracts for 72 h. Cell viability was then assessed using the MTT assay. The results showed that the BESD and BF1SD inhibited the proliferation of PC3 and MDA-MB-231 cells in a concentration-dependent manner (Figure 3 and Figure 4). The half-maximal inhibitory concentration (IC_50_) values determined for the BESD were 196.40 ± 6.20 µg/mL for PC3 cells and 182.40 ± 8.10 µg/mL for MDAMB-231 cells. In comparison, the IC_50_ values for the BF1SD were 281.10 ± 6.30 µg/mL for PC3 cells and 273.10 ± 3.10 µg/mL for MDA-MB-231 cells (Table 2).

### 2.5. Induction of Apoptosis by the BESD and BF1SD in PC3 Cells

**Figure 5:**
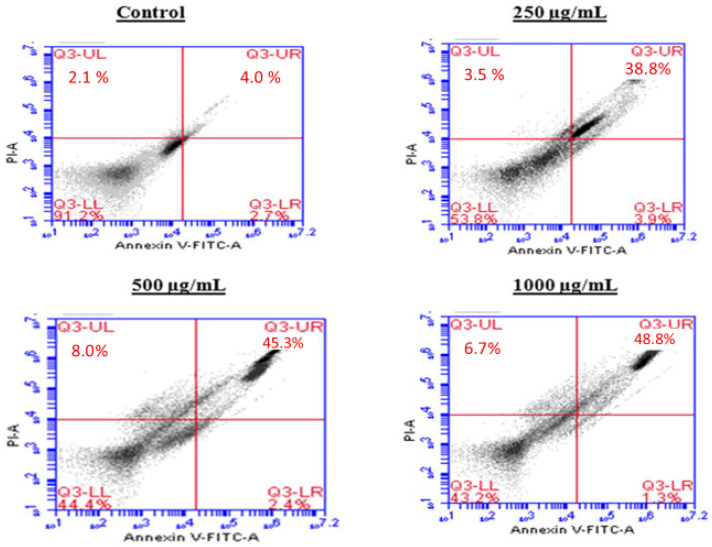
Induction of apoptosis by BESD in PC3 cancer cells. Representative histograms of cells sorted by flow cytometry. Cells were treated with the extract for 24 h at different concentrations (250, 500 and 1000 µg/mL) and stained with Annexin V-FITC and propidium iodide before analysis. The cells in quadrants Q3-UL, Q3-UR, Q3-LL, and Q3-LR represent necrotic, late apoptotic, viable, and early apoptotic populations, respectively.

**Figure 6:**
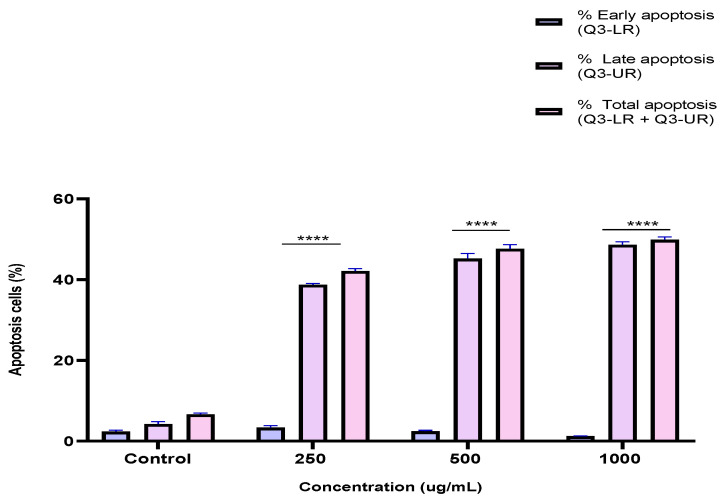
Induction of apoptosis by BESD in PC3 cancer cells. Quantification of apoptotic cells. The percentage of apoptotic cells was calculated. Each bar represents the mean ± standard deviation (n = 3), **** *p* < 0.0001, compared to the control. Two-way ANOVA analysis. BESD: *n*−butanolic extract of *Stachys duriaei*.

**Figure 7:**
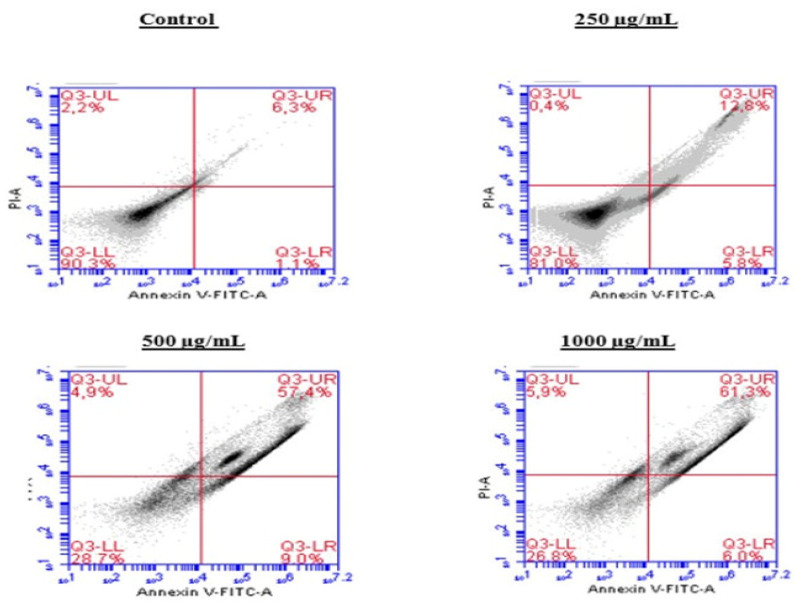
Induction of apoptosis by BF1SD in PC3 cancer cells. Representative histograms of cells sorted by flow cytometry. The cells were treated with the extract for 24 h at different concentrations (250, 500, and 1000 µg/mL) and stained with Annexin V-FITC and propidium iodide before analysis. The cells in quadrants Q3-UL, Q3-UR, Q3-LL, and Q3-LR represent necrotic, late apoptotic, viable, and early apoptotic populations, respectively.

**Figure 8:**
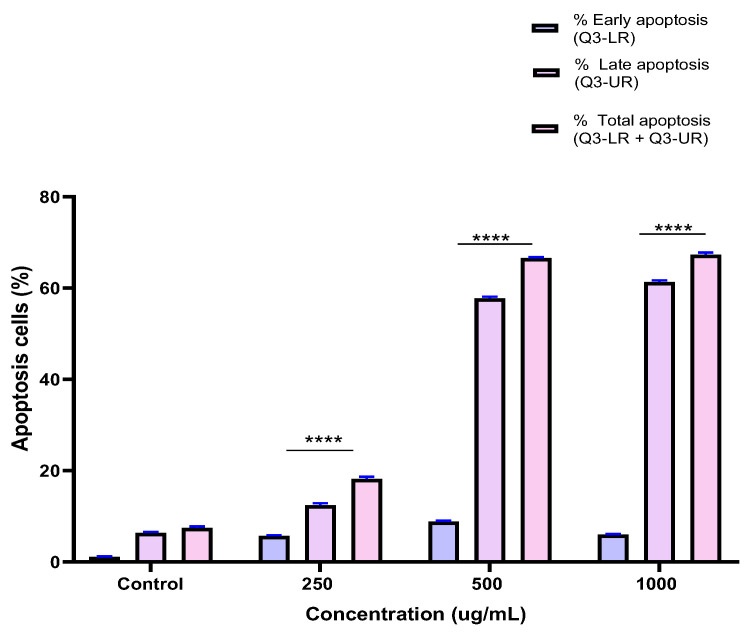
Induction of apoptosis by BF1SD in PC3 cancer cells. Proportion of apoptotic cells. The percentage of apoptotic cells was calculated. Each bar represents the mean ± standard deviation (n = 3), **** *p* < 0.0001, compared to the control. Two-way ANOVA analysis. BF1SD: First fraction of the *n*−butanolic extract of *Stachys duriaei*.

To investigate the mechanism by which the BESD and BF1SD inhibit cell proliferation, an apoptosis analysis was performed using flow cytometry with dual Annexin V-FITC/PI staining after a 24 h exposure to varying extract concentrations (250, 500, and 1000 µg/mL). The results indicated that the BESD and BF1SD induced apoptosis in PC3 cells, as evidenced by the accumulation of early and late apoptotic cells and a decrease in viable cells, with a more pronounced effect at 500 and 1000 µg/mL (*p* < 0.0001) compared to the negative control (Figure 5, Figure 6, Figure 7 and Figure 8). Following the BESD treatment, the total proportion of apoptotic cells (early and late) increased from 6.60 ± 0.30% (control) to 42.10 ± 0.60% (250 µg/mL), 47.70 ± 1.00% (500 µg/mL), and 49.90 ± 0.70% (1000 µg/mL). After the BF1SD treatment, the proportion of apoptotic cells increased from 7.50± 0.30% (control) to 18.20 ± 0.50% (250 µg/mL), 66.60 ± 0.20% (500 µg/mL), and 67.30 ± 0.50% (1000 µg/mL).

### 2.6. Induction of Cell Cycle Arrest in PC3 Cells by the BESD and BF1SD

**Figure 9:**
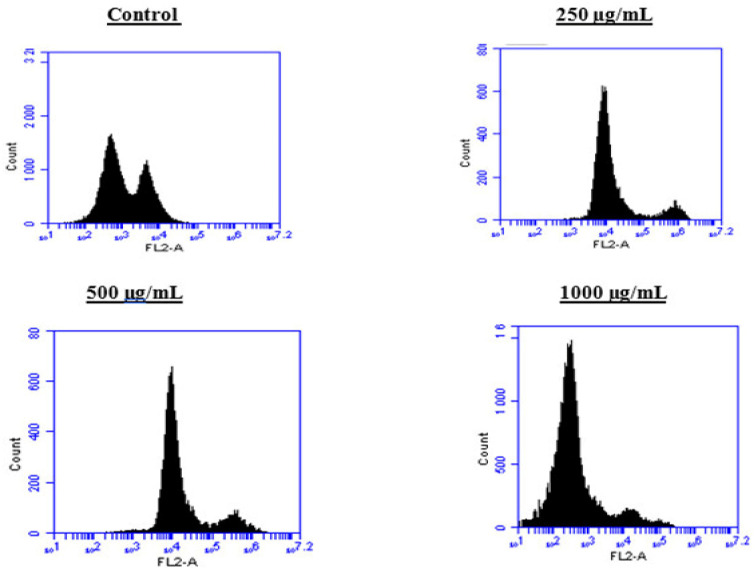
Effect of BESD on cell cycle distribution in PC3 cell lines. The PC3 cell line was incubated for 24 h with the extract at different concentrations (250, 500, and 1000 µg/mL). Flow cytometry analysis of cell distribution is represented by PI fluorescence histograms. The experiment was performed in triplicate.

**Figure 10:**
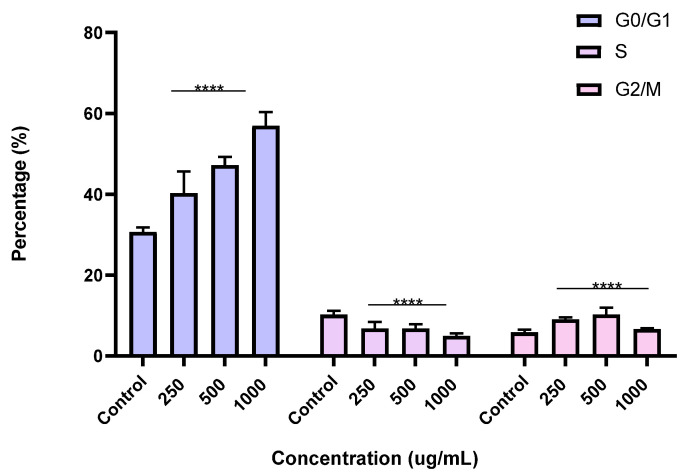
Effect of BESD on cell cycle distribution in PC3 cell lines. The PC3 cell line was incubated for 24 h with the extract at different concentrations (250, 500, and 1000 µg/mL). The percentages of cells in different phases are shown in the bar graph. BESD clearly affects the cell cycle by promoting accumulation in the G0/G1 phase. The effect is dose-dependent. Data are presented as mean ± standard deviation (n = 3), **** *p* < 0.0001, compared to the control. A two-way ANOVA was performed, followed by Bonferroni correction. BESD: *n*−butanolic extract of *Stachys duriaei*.

**Figure 11:**
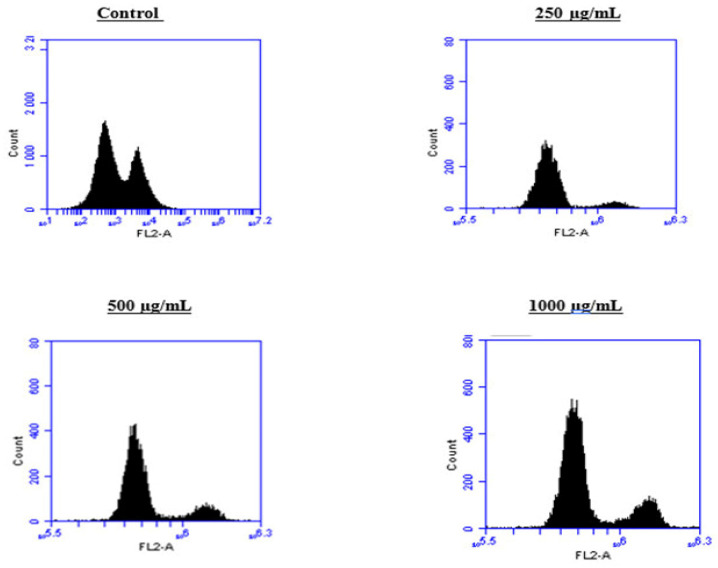
Effect of BF1SD on cell cycle distribution in PC3 cell lines. The PC3 cell line was incubated for 24 h with the extract at different concentrations (250, 500, and 1000 µg/mL). Flow cytometry analysis of cell distribution is represented by PI fluorescence histograms. The experiment was repeated three times.

**Figure 12:**
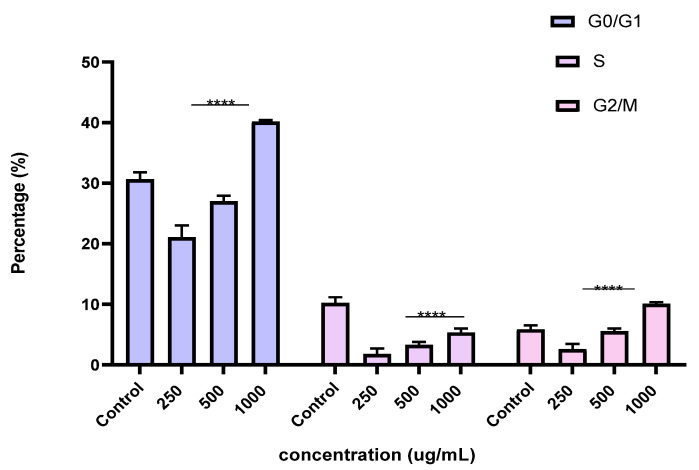
Effect of BF1SD on cell cycle distribution in PC3 cell lines. The PC3 cell line was incubated for 24 h with the extract at different concentrations (250, 500, and 1000 µg/mL). The percentages of cells in different phases are shown in the bar graph. BF1SD clearly affects the cell cycle by promoting accumulation in the G0/G1 and G2/M phases. The effect is dose-dependent. Data are presented as mean ± standard deviation (n = 3), **** *p* < 0.0001, compared to the control. A two-way ANOVA was performed, followed by Bonferroni correction. BF1SD: First fraction of the *n*−butanolic extract of *Stachys duriaei*.

To better understand the mechanism by which the BESD and BF1SD inhibit cell proliferation, a flow cytometry analysis using propidium iodide (PI) staining was performed to assess cell cycle distribution in the PC3 cells after 24 h of exposure to the extract at a concentration of 250, 500, and 1000 µg/mL. A 24 h treatment of PC3 cells with BESD led to significant concentration-dependent cell cycle modifications. Compared to the control (30.70 ± 1.10%), the G0/G1 phase increased to 40.30 ± 5.40% at 250 µg/mL, 47.20 ± 2.00% at 500 µg/mL, and 57.00 ± 3.40% at 1000 µg/mL. The S phase was markedly reduced compared to the control (10.20 ± 1.00%), reaching 6.80 ± 1.60% at 250 µg/mL, 6.80 ± 1.00% at 500 µg/mL, and 5.00 ± 0.60% at 1000 µg/mL. Meanwhile, the G2/M phase increased to 9.10 ± 0.50% at 250 µg/mL, 10.30 ± 1.70% at 500 µg/mL, and then decreased to 6.70 ± 0.20% at 1000 µg/mL. These results suggest that a high concentration (1000 µg/mL) of BESD induces cell cycle arrest at G0/G1, whereas at intermediate concentrations (250 and 500 µg/mL), it promotes transient accumulation of cells in G2/M, thereby delaying mitotic progression and limiting PC3 cell proliferation (Figure 9 and Figure 10). Similarly, the treatment of PC3 cells with the BF1SD for 24 h resulted in significant concentration-dependent cell cycle alterations. Compared to the control (30.70 ± 1.1%), the G0/G1 phase decreased to 21.10 ± 1.90% at 250 µg/mL, slightly increased to 27.00 ± 0.90% at 500 µg/mL, and significantly rose to 40.20 ± 0.30% at 1000 µg/mL. The S phase was drastically reduced compared to the control (10.2 ± 1.0%), reaching 1.80 ± 0.90% at 250 µg/mL, 3.30 ± 0.50% at 500 µg/mL, and 5.30 ± 0.70% at 1000 µg/mL. Meanwhile, the G2/M phase decreased to 2.60± 0.90% at 250 µg/mL, remained close to the control (5.90 ± 0.70%) at 500 µg/mL (5.60 ± 0.50%), but significantly increased to 10.10 ± 0.30% at 1000 µg/mL. These findings suggest that at a high concentration (1000 µg/mL), BF1SD induces dual cell cycle arrest at G0/G1 and G2/M, while at a low concentration (250 µg/mL), it inhibits cell cycle progression, thereby limiting PC3 cell proliferation (Figure 11 and Figure 12).

### 2.7. Ex Vivo Studies: Vascular Reactivity

#### 2.7.1. Vascular Reactivity to Acetylcholine (ACh)

**Figure 13:**
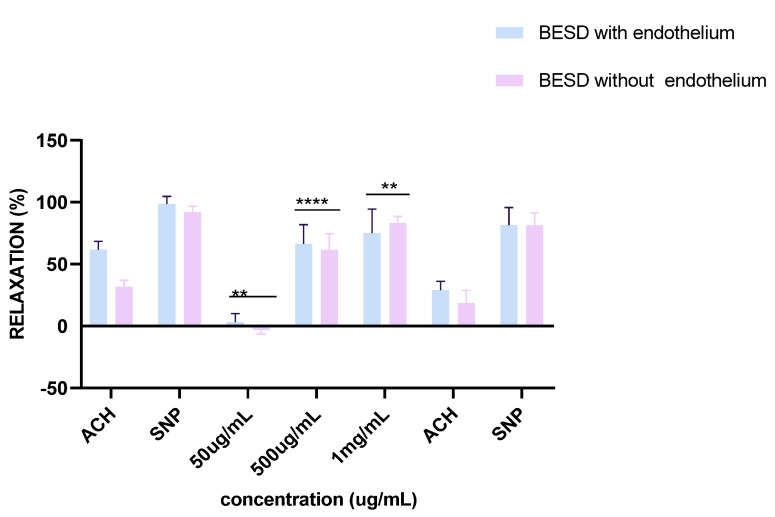
Percentage of relaxation induced by BESD at concentrations of 50 µg/mL, 500 µg/mL, and 1 mg/mL on rat aortic rings precontracted with Phe, with or without endothelium, after 15 min. **** *p* < 0.0001; with endothelium vs. without endothelium. BESD: *n*−butanolic extract of *Stachys duriaei*.

**Figure 14:**
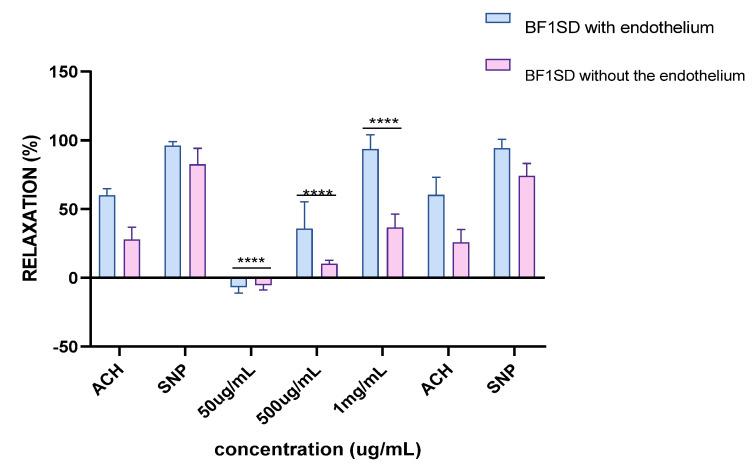
Percentage of relaxation induced by BF1SD at concentrations of 50 µg/mL, 500 µg/mL, and 1 mg/mL on rat aortic rings precontracted with Phe, with or without endothelium, after 15 min. **** *p* < 0.0001; with endothelium vs. without endothelium. BF1SD: First fraction of the *n*−butanolic extract of *Stachys duriaei.*

Experiments on vascular reactivity to acetylcholine revealed a marked dependence on the presence of the endothelium for both tested extracts, namely BESD and BF1SD. The BESD showed a strong endothelium dependence, although moderate vasorelaxant activity was also observed in its absence, reaching +15% relaxation at 1 mg/mL. In the presence of endothelium, it induced a pronounced dose-dependent response, with +80% relaxation at 500 µg/mL and +100% at 1 mg/mL, slightly exceeding that of the BF1SD (Figure 13). As for the BF1SD, a significant vasorelaxant response was observed only in the presence of the endothelium, with a pronounced dose-dependent increase at 500 µg/mL and 1 mg/mL (+75% and +95% relaxation, respectively). However, in the absence of the endothelium, the relaxation was almost abolished (less than +5% at all concentrations). These results suggest that BF1SD mainly acts via endothelium-dependent mechanisms, notably by stimulating the release of NO (Figure 14). These results highlight the superior overall efficacy of the BESD compared to the BF1SD while also confirming that its effect is mainly dependent on endothelium-based mechanisms.

#### 2.7.2. Vascular Reactivity to Sodium Nitroprusside (SNP)

Responses to sodium nitroprusside (SNP), a direct donor of nitric oxide (NO), were similar for both extracts, regardless of the presence or absence of the endothelium. Complete vasorelaxation was observed, reaching nearly +100% in both experimental conditions (with or without endothelium). These observations confirm that the vascular smooth muscle retains its normal reactivity and validate the integrity of the tissues used in these experiments.

## 3. Discussion

Cardiovascular diseases and cancer are the leading causes of mortality worldwide. A significant proportion of the currently used antitumor agents in clinical settings are derived from natural sources, highlighting the importance of natural products in oncology research [12]. In addition, many epidemiological studies indicate that a diet rich in fruits and vegetables helps to prevent cardiovascular diseases and reduce the risk of heart events, an effect primarily attributed to their flavonoid content, a class of polyphenols known for their antioxidant and vasodilatory properties [13]. Natural products are a preferred source of new therapeutic molecules, particularly for the treatment of cancer and cardiovascular diseases. Among the mechanisms of action of bioactive plant-derived compounds, we find the induction of apoptosis, cell cycle arrest, and modulation of vascular reactivity [14,15]. The genus *Stachys* holds potential therapeutic interest in oncology, notably due to its antiproliferative activity and anti-inflammatory properties. Plants of this genus are also known for their wound-healing, astringent, anti-diarrheal, anti-inflammatory, and antioxidant effects [4,7,8,9,16].

In this study, we evaluated the biological effects of BESD and BF1SD extracts derived from *Stachys duriaei*, highlighting their antiproliferative, pro-apoptotic, cell cycle-regulating, and vasorelaxant potential. The results obtained were correlated with the chemical composition of the extracts as analyzed by LC-HRMS/MS.

### 3.1. Antitumoral Activity (Antiproliferative—Induction of Apoptosis—Induction of Cell Cycle Arrest)

Our results show that the BESD and the BF1SD significantly inhibit the proliferation of PC3 and MDA-MB-231 cells in a concentration-dependent manner, with BESD exhibiting a stronger antiproliferative activity (IC_50_:196.40 ± 6.20 µg/mL for PC3 and 182.40 ± 8.10 µg/mL for MDA-MB-231) than the BF1SD (IC_50_:281.10 ± 6.30 µg/mL for PC3 and 273.10 ± 3.10 µg/mL for MDA-MB-231). This activity may be attributed to the higher antioxidant polyphenol content in the BESD, mainly flavonoids and phenolic acids, which have the ability to inhibit cell proliferation by modulating signaling pathways associated with carcinogenesis, as suggested by several studies [17,18]. Apoptosis, an essential cellular process for maintaining tissue homeostasis, plays a key role in regulating cell growth and preventing tumor proliferation [19]. In our study, BESD and BF1SD demonstrated a significant ability to induce apoptosis in PC3 cells. Apoptosis induction was confirmed by an increase in early and late apoptotic cells and a reduction in viable cells, particularly at concentrations of 500 and 1000 µg/mL. The results revealed that BESD induces a progressive apoptosis, with an increasing percentage of apoptotic cells at increasing concentrations, while BF1SD exhibits a more pronounced effect at higher concentrations. The cell cycle is a fundamental process in cell division and proliferation and represents a strategic target in the development of anticancer therapies. Tumor cells, characterized by uncontrolled proliferation, often exhibit dysfunctions in the regulatory mechanisms of the cell cycle. Therefore, inhibiting these mechanisms is a promising therapeutic approach to limit tumor progression [20]. Our results show that the BESD induces cell cycle arrest specifically at the G2/M phase, thereby blocking cell division and preventing tumor proliferation. In contrast, the BF1SD extract exhibits a more complex action by inducing a dual blockade at both the G0/G1 and G2/M phases, suggesting a broader modulation of cell cycle regulators. These effects are likely due to the presence of specific bioactive metabolites in each extract.

The metabolites present in the BESD and the BF1SD play a key role in inhibiting cell proliferation, modulating inflammatory pathways, inducing apoptosis, and causing cell cycle arrest. The differences observed between the two extracts can be attributed to their chemical compositions, particularly the specific presence of flavonoids and phenolic acids. These notable metabolites are described below.

Hyperoside, which represents 11.41% in the BESD and 5.00% in the BF1SD, stands out for its antiproliferative activity by regulating the PI3K/AKT and MAPK pathways. This compound has demonstrated in vivo efficacy by reducing tumor burden in DMBA/TPA-induced skin cancer models [21]. Moreover, its inhibitory effect on the STAT3 pathway and its antioxidant properties in human endothelial cells further enhance its therapeutic potential [22]. Hyperoside has been shown to inhibit the proliferation of A549 non-small cell lung carcinoma cells by triggering mitochondrial apoptosis, with caspase activation and modulation of signaling pathways such as JNK and p38 MAPK [23]. In breast cancer cells, hyperoside has been reported to modulate intracellular ROS levels and inhibit the NF-κB pathway, leading to a decrease in the expression of anti-apoptotic genes such as XIAP and Bcl-2, while promoting the accumulation of Bax, a pro-apoptotic factor [24]. This metabolite has also shown the ability to induce G1 phase cell cycle arrest in liver cancer cells by modulating the expression of key proteins involved in cell cycle regulation [25]. Furthermore, it has been associated with cell cycle inhibition and moderate apoptosis induction in bladder cancer cells through the activation of MAPK and Akt signaling pathways [26].

Luteolin-7-*O*-β-D-glucoside, present at 5.27% in the BESD and 4.64% in the BF1SD, inhibits tumor proliferation by blocking the activation of the NF-κB transcription factor and modulating cyclin D1 expression, thereby slowing cell cycle progression [27]. It plays a dual role, inducing apoptosis in cancer cells while protecting normal cells under stress conditions. It acts independently of caspases in certain cancer cells, such as HepG2 hepatocellular carcinoma cells, where it induces G2/M phase cell cycle arrest and promotes the nuclear translocation of apoptosis-inducing factor (AIF), suggesting a mitochondrial mechanism independent of caspases [28]. Conversely, in normal cells, it exerts a protective effect by reducing apoptosis through MAPK pathway modulation, specifically decreasing the phosphorylation of ERK1/2, JNK, and p38 while increasing ERK5 phosphorylation [29]. Additionally, this compound has demonstrated a significant ability to inhibit nasopharyngeal cancer cell proliferation by inducing cell cycle arrest at the S and G2/M phases. This inhibition is accompanied by chromatin condensation and activation of apoptotic processes, associated with the modulation of regulatory proteins in both intrinsic and extrinsic apoptotic pathways, as well as mitochondrial membrane potential depolarization [30].

Hydroxysafflor Yellow A (HSYA), present at 8.49% in BESD and 8.07% in BF1SD, exhibits remarkable antiproliferative properties by inhibiting the proliferation, migration, and invasion of colorectal cancer cells through the PPARγ/PTEN/Akt signaling pathway. It induces apoptosis in MCF-7 breast cancer cells by increasing ROS levels and regulating pro-apoptotic proteins such as Bax and p53 while reducing Bcl-2 and cyclin D1 expression and activating caspase-3. Additionally, HSYA inhibits the nuclear translocation of NF-κB/p65, suggesting suppression of the NF-κB pathway [31]. Another study highlighted its role in inhibiting the growth of hepatic cancer cells by blocking autophagic flux and inhibiting the PI3K/AKT/mTOR pathway. It has also demonstrated significant effects on cell cycle regulation, particularly in the S phase, by reducing the expression of cyclins D1 and E, as well as CDK2. Furthermore, this metabolite inhibits the PI3K/AKT pathway in MCF-7 breast cancer cells by reducing the expression of proteins such as p-PI3K, PI3K, AKT, and p-AKT, thereby limiting their proliferation [32].

Rutin, present at 3.66% in BESD and 4.58% in BF1SD, stands out for its antiproliferative activity by influencing the expression of DR4/DR5, Akt, ERK, and NF-κB proteins. It promotes apoptosis in various types of carcinomas, particularly in lung cancer [33]. Additionally, it enhances the effectiveness of conventional chemotherapy treatments such as 5-fluorouracil (5-FU) and oxaliplatin, offering a promising therapeutic synergy against colon cancer cells [34]. Rutin also exerts a significant apoptotic effect on human glioma cells by regulating the P53 protein. This apoptotic process is associated with increased ROS levels, loss of mitochondrial membrane potential, cytochrome c release, and activation of caspases-9 and -3. P53 suppression reversed rutin-induced apoptosis, confirming its key role in this mechanism [35]. Moreover, rutin has been identified as an agent that induces G2/M phase arrest in human neuroblastoma cell lines. This inhibition is accompanied by the modulation of pro- and anti-apoptotic proteins BAX and BCL-2, a reduction in MYCN mRNA expression, and a decrease in TNF-α secretion, suggesting a direct impact on tumor growth and cell survival [36].

Myricitrin, present at 6.50% in the BESD and 2.34% in BF1SD, has demonstrated significant antiproliferative activity against human HL-60 leukemia cells and exhibits anti-angiogenic properties, suggesting its potential role in preventing the formation of new blood vessels essential for tumor growth [37]. It exerts a protective effect against apoptosis in normal cells, particularly in myocardial cells, while inducing apoptosis in colorectal cancer cells via the PI3K/AKT/mTOR pathway [38]. Furthermore, myricitrin has been shown to inhibit the proliferation of gastric cancer cells by inducing cell cycle arrest and activating apoptotic pathways. Its effect has also been observed in hepatocellular carcinoma cells, where it induces G2/M phase arrest by modulating signaling pathways involved in cell cycle progression [39,40].

Peltatoside (5.07% in BESD, 2.19% in BF1SD) and sayaendoside (5.89% in BESD, 6.62% in BF1SD) also exhibit significant anti-inflammatory properties by modulating the immune response and reducing leukocyte recruitment, which could contribute to a tumor environment less favorable for cancer growth [41,42].

Other metabolites, such as Naringenin-7-*O*-β-D-glucoside and kaempferol-3-O-rutinoside, exert antiproliferative effects by targeting key signaling pathways involved in cell proliferation regulation, such as PI3K/Akt and MAPK/ERK. Their inhibition contributes to reduced tumor growth, enhanced autophagy, and inhibition of tumor progression, particularly in gastric cancers [43]. These compounds also promote apoptosis through mechanisms dependent on PI3K/Akt and MAPK/ERK signaling modulation. Specifically, Naringenin-7-*O*-β-D-glucoside induces dose-dependent cytotoxicity in MDA-MB-231 triple-negative breast cancer cells, accompanied by increased DNA fragmentation and reduced expression of the epidermal growth factor receptor (EGFR), suggesting interference with tumor progression pathways [44]. Kaempferol-3-O-rutinoside, in turn, inhibits the proliferation of human breast cancer cells by activating AMPK, leading to tumor growth suppression and apoptosis induction [45].

### 3.2. Vascular Reactivity

Ex vivo analyses performed on aortic rings revealed that the BESD and the BF1SD induced endothelium-dependent vasorelaxation. BESD and BF1SD allowed complete or near-complete aortic relaxation (100% and 95%, respectively) at a concentration of 1 mg/mL. In deendothelialized vessels, the relaxing effects of both extracts were significantly reduced (<15% relaxation), indicating that the extracts exert their relaxing action through endothelium-dependent mechanisms. A likely mechanism accounting for the observed BESD- and BF1SD-induced vasorelaxation is the release of nitric oxide (NO), a key factor in the regulation of vascular function [46]. Indeed, phenolic and flavonoid compounds detected in both extracts have been shown to stimulate NO production and to induce relaxation of vascular smooth muscles.

Hyperoside has demonstrated an endothelium-dependent vasorelaxant effect. Previous studies [31] have demonstrated that hyperoside promotes NO production by the endothelium, thereby inducing relaxation of vascular smooth muscles. Hyperoside has been reported to induce concentration-dependent vasorelaxation in isolated coronary arteries, with this effect being partially inhibited by blocking NO synthesis [47,48].

Luteolin-7-*O*-β-D-glucoside has also demonstrated vasorelaxant effects, primarily acting through an NO-dependent pathway. Li (2019) reported that Luteolin-7-*O*-β-D-glucoside stimulates NO production in coronary arteries, contributing to vasorelaxation [49]. Significant vasorelaxation has been observed in isolated rat arteries, indicating that this effect is mediated by the NO-dependent pathway. Furthermore, studies have suggested that Luteolin-7-*O*-β-D-glucoside could influence ion channels in vascular smooth muscle cells, particularly ATP-sensitive potassium (K_ ATP) channels, leading to cellular hyperpolarization and vasorelaxation [50].

Hydroxysafflor Yellow A (HSYA) also demonstrated a significant vasorelaxant effect, acting through an endothelium-dependent mechanism. Li (2014) showed that HSYA induced vasorelaxation through NO release in isolated coronary arteries, supporting its role in modulating endothelial function [31].

Rutin also exhibited marked vasorelaxant properties, likely acting by activating K ATP channels. Adaramoye (2009) observed that rutin promotes vasorelaxation by inducing hyperpolarization of smooth muscle cells, leading to relaxation of blood vessels [51]. Myricitrin showed a dose-dependent vasorelaxant effect, primarily by stimulating NO release and activating K ATP channels in vascular smooth muscle cells. Si (2014) observed such an effect in rat arteries, confirming the involvement of these mechanisms in vasorelaxation [48].

Finally, Kaempferol-3-*O*-rutinoside also showed the ability to promote NO production in the endothelium of rat coronary vessels, inducing vasorelaxation [52].

Overall, these results suggest that the vasorelaxant effect of BESD and BF1SD extracts is primarily mediated by NO production, with modulation of ion channels such as K ATP channels through an endothelium-dependent mechanism. These findings underscore the therapeutic potential of these extracts in regulating vascular function.

## 4. Materials and Methods

### 4.1. Plant Material

The plant *Stachys duriaei* de Noé was collected in May 2019 in the Constantine region, specifically in El Kantour (coordinates: x = 6.750028, y = 36.573333, z = 521 m). Its identification was carried out by Prof. Gérard de Belair (ENSA, El Harrach, Algeria), and it is listed in the herbarium of the LOST Laboratory at the University of Mentouri Constantine under the voucher code 099_41. It is also recorded in the national herbarium GdB under the code 099_41.

The aerial parts of *Stachys duriaei* (1.5 kg), previously dried and ground, were macerated in a hydroethanolic mixture (ethanol/water: 80/20, *v*/*v*) for 3 × 24 h. The crude extracts obtained were redissolved in distilled water (1000 mL) and then subjected to liquid–liquid extractions using solvents of increasing polarity (petroleum ether, chloroform, ethyl acetate, and *n*-butanol). The resulting organic phases were concentrated under reduced pressure until dryness to obtain the respective extracts: PESD (0.8 g), CESD (1.6 g), EAESD (2.5 g), and BESD (10 g).

### 4.2. Fractionation of the n-Butanolic Extract of Stachys duriaei by Vacuum Liquid Chromatography (VLC)

BESD (5 g) was fractionated by vacuum liquid chromatography (VLC) using SC6 polyamide gel as the stationary phase and a toluene-methanol mixture with different gradients (100/0, 80/20, 75/25, 70/30, 65/35, 60/40, 40/60, 20/80, and finally 0/100) as the mobile phase. Nine fractions were collected for each mixture and analyzed by thin-layer chromatography (TLC). TLC monitoring under UV light revealed complex mixtures, which were grouped into three main fractions.

### 4.3. Sample Preparation

The BESD and the VLC fractions of the *n*-butanolic extract (BF1SD) of *Stachys duriaei* were dissolved in dimethyl sulfoxide (DMSO, Sigma Aldrich, St. Louis, MO, USA), then completed with RPMI medium. The DMSO content in the solution did not exceed 0.1%. The mixture was then centrifuged to remove insoluble compounds, and the supernatant was filtered using sterilizing filters. The obtained solution was diluted in RPMI medium and prepared at different concentrations.

### 4.4. LC-HRMS/MS Analyses (Liquid Chromatography—High Resolution Tandem Mass Spectrometry)

The LC-ESI-DDA-HRMS/MS spectra were obtained using a Dionex Ultimate 3000 (HPLC), Thermo Fisher Scientific, Sunnyvale, CA, USA liquid chromatography system coupled with a Thermo Instruments mass spectrometer (LTQ Orbitrap XL). The HPLC analysis was performed on a Waters BEH C18 column (1.7 µm, 2.1 mm × 150 mm) using the following solvents: Solvent A (water with 0.1% formic acid) and Solvent B (acetonitrile with 0.1% formic acid). The applied elution gradient was 5% B for 5 min, from 5% to 100% over 20 min, then 100% for 7 min. The flow rate used was 0.150 mL/min, and the column temperature was maintained at 45 °C. MS/MS spectra were obtained using the MZmine 2.53 software, with a mass noise detection threshold set at 2E4 and 2E for positive and negative ion modes. Compound identification was facilitated using the Global Natural Product Social Molecular Networking (GNPS) platform (https://gnps.ucsd.edu/).

### 4.5. Cell Culture

The MDA-MB-231 cells, derived from a pleural effusion of a mammary adenocarcinoma and obtained from ATCC, were cultured in DMEM medium (PAN Biotech, Aidenbach, Germany) supplemented with 10% fetal bovine serum (FBS; Dominique Dutscher, SA, Brumath, France) and 1% penicillin/streptomycin. The PC3 cell line, derived from prostate cancer and obtained from ATCC, was cultured in RPMI 1640 medium (PAN Biotech, Aidenbach, Germany) supplemented with 10% FBS and 1% penicillin/streptomycin. The cell lines were maintained in an incubator at 37 °C with an atmosphere containing 5% CO_2_ and 95% humidity.

### 4.6. MTT Assay

The antiproliferative activity of the BESD and BF1SD extracts on the two selected cell lines was evaluated by the MTT assay (3-[4,5-dimethylthiazol-2-yl]-2,5-diphenyltetrazolium bromide; Sigma-Aldrich), according to the method of Mossman 1983 [53]. PC3 and MDA-MB-231 cells (5000 cells/well) were seeded in 96-well plates and incubated with different concentrations of BESD (0.85, 2.54, 7.62, 22.86, 68.59, 205.8, 617.3, 1851.8, 5555.5, 16,666.6, 50,000 µg/mL) and BF1SD (19.53, 39.06, 78.25, 156.25, 312.5, 625, 1250, 2500, 5000, 10,000, 20,000 µg/mL). The concentration ranges of the *Stachys duriaei* extracts were intentionally broad in order to account for the complexity of the plant matrix and the lack of previous data, with interpretation based solely on the concentrations that did not show precipitation or optical interferences. Untreated cells served as controls. After 72 h of exposure to the extracts, the wells were emptied, washed with PBS, and then 100 µL of MTT (5 mg/mL) was added to each well. The plates were then incubated for an additional 2 h at 37 °C with 5% CO_2_. After this incubation, the medium was carefully aspirated, and the formed formazan crystals were dissolved in DMSO. The absorbance of each well was measured at 570 nm using a microplate reader (Varioskan LUX, Thermo Scientific).

### 4.7. Apoptosis Assay

Cellular apoptosis induced by the BESD and BF1SD was detected by Annexin V-FITC/PI staining, following the manufacturer’s instructions (BD Pharmingen™ FITC Annexin V Apoptosis Detection Kit). PC3 cells (10^5^ cells/well) were treated with BESD and BF1SD after seeding in 96-well plates at different concentrations (250, 500, and 1000 µg/mL) for 24 h. The cells were then washed with PBS, centrifuged, and stained with 5 µL of Annexin V and 5 µL of propidium iodide (PI). After a 15 min incubation at room temperature, 400 µL of binding buffer was added. The labeled cells were analyzed by flow cytometry using the Accuri™ TM-C6 (BD Biosciences, San Jose, CA, USA).

### 4.8. Cell Cycle Analysis

The cell cycle was determined using a cell cycle detection kit (BD Pharmingen™ PI/RNase Staining Buffer). PC3 cells (10^6^ cells/well) were treated with the BESD and BF1SD after seeding in 6-well plates at different concentrations (250, 500, and 1000 µg/mL) for 24 h. At the end of the treatment, the cells were collected, washed with cold PBS, and then fixed in cold 70% ethanol (−20 °C) for 2 h. After centrifugation, the cell pellet was resuspended in 0.5 mL of a mixture containing propidium iodide and RNase. After 15 min of incubation at room temperature, the DNA content of the cells was quantified by flow cytometry using the Accuri™ TM-C6 (BD Biosciences).

### 4.9. Ex Vivo Studies: Vascular Reactivity

Experimental protocols were performed in an accredited laboratory (UMR UGA—Inserm U1039 Radiopharmaceutiques BIocliniques, agreement # E3851610005) and were approved by the local ethical committee (Cometh, C2EA #12, Apafis authorization # 38828).

The experiments were conducted on male Sprague Dawley rats weighing between 350 and 400 g. The rats were euthanized using CO_2_. The chest cavity was opened, and the thoracic aorta was harvested. The vessel was placed in a modified Krebs solution and cleared of all connective tissue. The aorta was cut into 3–4 mm rings. These rings were placed in 15 mL organ baths and suspended on force transducers under a tension of 2 g. Each bath contained 15 mL of oxygenated Krebs physiological solution at 37 °C. The endothelium was preserved in some rings and mechanically damaged in others (for this, aortic rings underwent mechanical denudation of the endothelium). The equilibration period before each experiment was 1.5 h. At the end of the equilibration, the rings were stimulated with phenylephrine (10^−6^ M) and relaxed with acetylcholine (10^−6^ M) to verify the integrity of the endothelium and then relaxed with sodium nitroprusside (10^−5^ M) to check the vessel integrity. After confirming the integrity of a ring, two groups were made, with or without endothelium. The rings were then contracted with phenylephrine (10^−6^ M), and increasing cumulative doses of BESD and BF1SD were tested (50 µg/mL, 500 µg/mL, 1 mg/mL). Finally, a second test of integrity was performed to check the integrity of the vasomotor functions.

### 4.10. Statistical Analyses

All experiments were carried out in triplicate. Data analysis was performed using GraphPad PRISM software (version 8.0.2). Data are expressed as mean ± standard deviation (SD). A two-way analysis of variance (ANOVA), followed by the Bonferroni post hoc test, was used. Significant differences were defined as a *p*-value < 0.05.

## 5. Conclusions

The results obtained in this study highlight the biological potential of the *n*-butanolic extract (BESD) and a VLC fraction of the *n*-butanolic extract (BF1SD) of *Stachys duriaei*, particularly through their antiproliferative activity, ability to induce apoptosis, cell cycle arrest, and endothelial-dependent vasorelaxant effect. Phytochemical analysis identified a diversity of bioactive metabolites, mainly flavonoids and phenolic acids, whose biological properties are well-documented in scientific literature. The inhibition of cell proliferation observed in PC3 and MDA-MB-231 cancer cell lines suggests a potential role of these extracts in modulating signaling pathways involved in carcinogenesis. Furthermore, the observed induction of apoptosis and alteration of the cell cycle strengthen the hypothesis that these compounds could form a basis for the development of anticancer therapeutic strategies. Moreover, ex vivo tests on vascular reactivity revealed a vasodilatory effect of the extracts, highlighting their possible involvement in regulating endothelial functions and preventing cardiovascular diseases. This activity seems to be mediated by the release of nitric oxide (NO), a key mechanism in modulating blood pressure and vascular homeostasis. Overall, our results open promising prospects for the exploitation of *Stachys duriaei* extracts as sources of bioactive molecules of pharmacological interest. However, further investigations, particularly in vivo studies and clinical trials, are needed to deepen the understanding of the underlying mechanisms of action and assess their potential applications in oncology and cardiology.

## Figures and Tables

**Table 1:** LC-RHMS/MS analysis of compounds present in the *n*−butanolic extract of *Stachys duriaei* (BESD) and the first fraction of the *n*−butanolic extract of *Stachys duriaei* (BF1SD).

No	Compounds	Rt (Min)	*m/z* (Molecular Ion)	Intensity BESD	Intensity BF1SD
1	2′,3′-Epoxyindicolactone	4.14	383.1147	50,238,147 (1.01%)	233,411,187 (5.06%)
2	Melibiose	4.35	365.1042	45,638,908 (0.91%)	135,487,001 (2.94%)
3	*Trans* 4-*O*-Caffeoylquinic acid	7.41	353.1073	5,454,199 (0.11%)	97,332,808 (2.11%)
4	Adenosine	10.24	268.1030	1,613,449 (0.03%)	33,593,786 (0.73%)
5	4-*p*-Coumaroylquinic acid	17.31	339.1067	920,113 (0.02%)	2,140,260 (0.05%)
6	Chlorogenic acid	17.60	355.1015	16,240,683 (0.33%)	6,546,218 (0.14%)
7	Aloesin	17.85	395.1325	1,033,557 (0.02%)	3,631,532 (0.08%)
8	*N*-(6-methoxyquinolin-8-yl)alanine	18.23	247.1071	108,964 (0.00%)	682,061 (0.01%)
9	8-[5-(5,7-Dihydroxy-4-oxo-chromen-2-yl)-2-methoxy-phenyl]-5-hydroxy-2-(4-hydroxyphenyl)-7-methoxy-chromen-4-one	18.60	397.1470	18,970,320 (0.38%)	31,610,243 (0.69%)
10	1,2,3,4-Tetrahydro-β-carboline-3-carboxylic acid	18.99	231.1136	11,824,363 (0.24%)	16,993,092 (0.37%)
11	Vicenin 2	19.13	595.1651	1,233,768 (0.02%)	619,904 (0.01%)
12	3-*p*-Coumaroylquinic acid	19.20	339.1068	920,529 (0.02%)	7,626,802 (0.17%)
13	*N*-methyl-2,4-dihydroxy-3-phenylquinoline	19.37	252.0863	14,777,481 (0.30%)	67,508,138 (1.46%)
14	1,2,6-Trigalloylglucose	19.53	635.0864	107,962,646 (2.16%)	3,546,009 (0.08%)
15	Methyl chlorogenate	19.59	369.1172	2,114,685 (0.04%)	13,688,373 (0.30%)
16	1,6-bis-*O*-galloyl-β-D-glucose	19.70	483.0758	251,605,789 (5.04%)	8,383,446 (0.18%)
17	Myricetin-3-rutinoside	19.75	627.1544	98,083,034 (1.90%)	17,940,256 (0.39%)
18	8-*O*-Acetylharpagide	19.78	429.1359	12,707,623 (0.25%)	39,216,825 (0.85%)
19	β-D-Glucosiduronic acid, 2-(3,4-dihydroxyphenyl) -5-hydroxy-6-methoxy-4-oxo-4H-1-benzopyran-7-yl	20.16	395.0939	27,823,579 (0.56%)	12,271,382 (0.27%)
20	2′-*O*-galloylhyperin	20.17	617.1126	256,823,533 (5.14%)	11,358,492 (0.25%)
21	Myricetin 3-galactoside	20.35	481.0964	143,761,867 (2.88%)	10,531,074 (0.23%)
22	Peltatoside (quercetin-3-*O*-arabinoglucoside)	20.59	597.1439	252,993,403 (5.07%)	101,001,688 (2.19%)
23	Sayaendoside	20.62	439.1569	294,250,466 (5.89%)	305,140,167 (6.62%)
24	Palatinose	20.84	325.0910	2,161,600 (0.04%)	734,945 (0.02%)
25	Hydroxysafflor yellow A	21.00	611.1595	423,917,474 (8.49%)	372,294,712 (8.07%)
26	Hyperoside	21.01	465.1015	569,496,692 (11.41%)	230,351,300 (5.00%)
27	β-D-Glucosyl 2-phenylethyl-6-*O*-β-D-xylopyranoside	21.14	439.1565	2,054,691 (0.04%)	11,365,070 (0.25%)
28	Rutin	21.15	611.1592	182,945,024 (3.66%)	211,206,590 (4.58%)
29	*N*-acetyltryptophan	21.25	247.1071	1,860,401 (0.04%)	31,686,457 (0.69%)
30	(*E*,*E*)-3-Methyl-5-(2,6,6-trimethyl-1-cyclohexen-1-yl)-2,4-pentadienoic acid	21.44	443.1894	2,362,093 (0.05%)	44,427,156 (0.96%)
31	Isovitexin	21.47	433.1119	11,963,439 (0.24%)	18,199,468 (0.39%)
32	Quercetin	21.62	303.0493	9,263,420 (0.19%)	6,573,430 (0.14%)
33	Naringenin-7-*O*-β-D-glucoside	21.63	435.1274	22,373,699 (0.45%)	138,909,348 (3.01%)
34	6′′-*O*-L-arabinopyranosyl astragalin	21.77	581.1489	2,508,029 (0.05%)	40,684,936 (0.88%)
35	kaempferol-3-*O*-rutinoside	21.78	595.1647	103,900,471 (2.08%)	118,492,531 (2.57%)
36	1,2,3,6-tetragalloylglucose	21.86	787.0966	231,807,467 (4.64%)	4,140,064 (0.09%)
37	Luteolin-7,3′-di-*O*-β-D-glucoside	22.12	609.1444	0 (0%)	193,827 (0.04%)
38	Pulvinic acid	22.15	307.0447	5,145,102 (0.10%)	34,943,156 (0.76%)
39	Luteolin-7-*O*-β-D-glucoside	22.16	449.1066	263,284,078 (5.27%)	213,769,931 (4.64%)
40	3’-Methoxyquercetin-3-*O*-rutinoside	22.33	625.1745	2,807,934 (0.06%)	32,042,186 (0.69%)
41	7-*O*-Neohesperidosyl chrysoeriol	22.38	609.1804	0 (0%)	196,837 (0.04%)
42	Roseoside	22.43	387.2001	6,909,671 (0.14%)	18,017,930 (0.39%)
43	1,3-Dihydroxyanthraquinone	22.59	239.0190	25,438,392 (0.51%)	47,110,390 (1.02%)
44	8,8-dimethyl-2-phenylpyrano[2,3-f]chromen-4-one	22.65	305.1125	272,436 (0.01%)	11,677,199 (0.25%)
45	β-Penta-*O*-galloyl-glucose	22.91	939.1073	53,919,155 (1.08%)	0 (0%)
46	Subaphyllin	23.01	314.1379	0 (0%)	870,665 (0.02%)
47	Tiliroside	23.04	593.1490	485,708 (0.01%)	723,658 (0.02%)
48	Myricetin-3-*O*-β-D-galactoside 6′′-*O*-gallate	23.18	631.0919	118,510,736 (2.37%)	8,806,699 (0.19%)
49	L-phenylalanine butyl ester	23.27	222.1483	332,365,866 (6.66%)	90,019,584 (1.95%)
50	Byzantionoside B	23.72	373.2196	13,907,102 (0.28%)	92,036,025 (2.00%)
51	(2*R*,3*S*,4*S*,5*R*,6*S*)-2-(hydroxymethyl)-6-[4-(hydroxymethyl)-1-propan-2-ylcyclohex-3-en-1-yl]oxyoxane-3,4,5-triol	23.85	355.1718	268,591,559 (5.38%)	327,376,033 (7.10%)
52	Diosmin	24.36	607.1649	0 (0%)	273,383 (0.01%)
53	Nematophin	24.66	273.1648	3,474,799 (0.07%)	1,634,480 (0.04%)
54	Luteolin	24.68	287.0543	4,595,397 (0.09%)	9,688,778 (0.21%)
55	4′-Methylisoscutellarein-7-[2-*O*-(6-*O*-acetyl-β-D-allopyranosyl)-β-D-glucoside	25.03	667.1860	3,509,386 (0.07%)	178,674,894 (3.88%)
56	9-(2,3-dihydroxypropoxy)-9-oxononanoic acid	25.22	261.1336	0 (0%)	582,638 (0.01%)
57	Apigenin-7-*O*-β-D-glucoside	25.36	431.0966	1,876,149 (0.04%)	1,578,292 (0.03%)
58	Myricitrin	25.85	463.0863	324,708,952 (6.50%)	108,056,798 (2.34%)
59	Geranyl-6-*O*-α-L-arabinofuranosyl-*O*-β-D glucoside	25.96	471.2197	18,719,776 (0.37%)	83,466,864 (1.81%)
60	(*R*)-Linalyl-β-vicianoside	26.20	471.2194	69,919,724 (1.40%)	250,642,158 (5.44%)
61	Naringenin	26.47	271.0604	61,307,962 (1.23%)	114,285,140 (2.48%)
62	8,3′,4′-trihydroxyflavone-7-*O*-(6′-*O*-*p*-coumaroyl)-β-D-glucoside	26.89	593.1493	117,608,434 (2.36%)	216,792,607 (4.70%)
63	2-butanone,4-[3-(β-D-glucopyranosyloxy)-4-hydroxy-2,6,6-trimethyl-1-cyclohexen-1-yl]	26.92	371.2043	4,451,363 (0.09%)	2,517,190 (0.05%)
64	6,8-dihydroxy-3-(10-hydroxyundecyl)-3,4-dihydroisochromen-1-one	26.98	351.2133	38,345,199 (0.77%)	71,191,586 (1.54%)
65	Narcissin	27.23	623.1595	19,122,741 (0.38%)	33,449,19 (0.73%)
66	Lauryldiethanolamide | *N,N*-bis(2-hydroxyethyl)dodecanamide	27.88	288.2525	450,500 (0.01%)	49,395,923 (1.07%)
67	Phytosphingosine	28.69	318.2994	0 (0%)	450,011 (0.01%)
68	Afzelin	28.77	431.0968	23,368,330 (0.47%)	27,836,625 (0.60%)
69	Emodin	28.85	269.0447	10,100,304 (0.20%)	7,322,516 (0.16%)
70	2′,4′,6′-Trihydroxydihydrochalcone	30.78	257.0811	204,060 (0.004%)	0 (0%)
71	Tetrahydroxyflavone	31.12	285.0394	14,040,475 (0.28%)	183,589,163 (3.98%)
72	Pyrenophorol	31.41	311.1673	2,104,127 (0.04%)	2,137,819 (0.05%)

BESD: *n*−butanolic extract of *Stachys duriaei*; BF1SD: First fraction of the *n*−butanolic extract of *Stachys duriaei*; RT: retention time.

**Table 2:** IC_50_ values (µg/mL) of the *n*-butanolic extract of *Stachys duriaei* (BESD) and the first fraction of the *n*-butanolic extract of *Stachys duriaei* (BF1SD) against the prostate cancer cell line (PC3) and the breast carcinoma cell line (MDA-MB-231).

	IC_50_ Values (μg/mL)	
Cell Type	BESD	BF1SD
PC3	196.40 ± 6.20	281.10 ± 6.30
MDA-MB-231	182.40 ± 8.10	273.10 ± 3.10

## Data Availability

All the data in the article are available from the corresponding author upon reasonable request.

## Supplementary Materials

The following supporting information can be downloaded at: https://www.mdpi.com/article/10.3390/molecules30173570/s1, Table S1: LC-RHMS/MS analysis of compounds present in the *n*-butanolic extract of *Stachys duriaei* (BESD) and the first fraction of the *n*-butanolic extract of *Stachys duriaei* (BF1SD).
