# Supplementary material for: LC-HRMS/MS-Guided Profiling and Biological Evaluation of Stachys duriaei Extracts: Anticancer and Vasorelaxant Mechanisms via Apoptosis and Endothelium-Dependent Pathways
Source: Molecules. 2025 Aug 31;30(17):3570. doi: 10.3390/molecules30173570 (PMC12430585; doi:10.3390/molecules30173570)
Supplement: Supplementary file 1 [file molecules-30-03570-s001.zip › molecules-3747555-supplementary.pdf]

Table S1. LC-RHMS/MS analysis of compounds present in the *n*-butanolic extract of *Stachys duriaei* (BESD) and the first fraction of the *n*-butanolic extract of *Stachys duriaei* (BF1SD).

No	SMILE	CAS	Compounds	Rt (min)	*m/z* (molecular ion)	Intensity BESD	Intensity BF1SD
1	CC1=CC(OC1=O)CC2(C(O2)COC3=C4C(=CC5=C3OC=C5)C=CC(=O)O4)C	312619-44-6	2',3'-Epoxyindicolactone	4.14	383.1147	50 238 147 (1.01%)	233411 187 (5.06%)
2	C(C1C(C(C(C(O1)OCC2C(C(C(C(O2)O)O)O)O)O)O)O)O	5340-95-4	Melibiose	4.35	365.1042	45638 908 (0.91%)	135487 001 (2.94%)
3	C1C(C(C(CC1(C(=O)O)O)O)OC(=O)C=CC2=CC(=C(C=C2)O)O)O	905-99-7	*Trans* 4-*O*-Caffeoylquinic acid	7.41	353.1073	5 454 199 (0.11%)	97332 808 (2.11%)
4	C1=NC(=C2C(=N1)N(C=N2)C3C(C(C(O3)CO)O)O)N	58-61-7	Adenosine	10.24	268.1030	1613 449 (0.03%)	33593 786 (0.73%)
5	C1C(C(CC1(C(=O)O)O)O)OC(=O)C=C/C2=CC=C(C=C2)O)O	1108200-72-1	4-*p*-Coumaroylquinic acid	17.31	339.1067	920 113 (0.02%)	2 140 260 (0.05%)
6	C1C(C(C(CC1(C(=O)O)O)OC(=O)C=CC2=CC(=C(C=C2)O)O)O)O	202650-88-2	Chlorogenic acid	17.60	355.1015	16 240 683 (0.33%)	6 546 218 (0.14%)
7	CC1=CC(=C(C2=C1C(=O)C=C(O2)CC(=O)C)C3C(C(C(C(O3)CO)O)O)O)O	30861-27-9	Aloesin	17.85	395.1325	1033 557 (0.02%)	3 631 532 (0.08%)
8	COC1=CC(=C2C(=C1)C=CC=N2)N	90-52-8	*N*-(6-methoxyquinolin-8-yl)alanine	18.23	247.1071	108 964 (0.00%)	682 061 (0.01%)
9	COC1=C(C=C(C=C1)C2=CC(=O)C3=C(C=C(C=C3O2)O)O)C4=C(C=C(C5=C4OC(=CC5=O)C6=CC=C(C=C6)O)O)OC	41583-84-0	8-[5-(5,7-Dihydroxy-4-oxo-chromen-2-yl)-2-methoxy-phenyl]-5-hydroxy-2-(4-hydroxyphenyl)-7-methoxy-chromen-4-one	18.60	397.1470	18 970 320 (0.38%)	31610 243 (0.69%)
10	CC1C2=C(CC(N1)C(=O)O)C3=CC=CC=C3N2	5470-37-1	1,2,3,4-Tetrahydro-β-carboline-3-carboxylic acid	18.99	231.1136	11 824 363 (0.24%)	16993 092 (0.37%)
11	C1=CC(=CC=C1C2=CC(=O)C3=C(C(=C(C(=C3O2)C4C(C(C(C(O4)CO)O)O)O)O)C5C(C(C(C(O5)CO)O)O)O)O)O	23666-13-9	Vicenin-2	19.13	595.1651	1 233 768 (0.02%)	619 904 (0.01%)

12	C1C(C(C(CC1(C(=O)O)O)OC(=O)/C=C/C2=CC=C(C=C2)O)O)O	87099-71-6	3-*p*-Coumaroylquinic acid	19.20	339.1068	920 529 (0.02%)	7 626 802 (0.17%)
13	CN1C2=CC=CC=C2C(=C(C1=O)C3=CC=CC=C3)O	519-66-4	*N*-methyl-2,4-dihydroxy-3-phenylquinoline	19.37	252.0863	14 777 481 (0.30%)	67508 138 (1.46%)
14	C1=C(C=C(C(=C1O)O)O)C(=O)OCC2C(C(C(C(O2)OC(=O)C3=CC(=C(C(=C3)O)O)O)O)OC(=O)C4=CC(=C(C(=C4)O)O)O)O)O	79886-49-0	1,2,6-Trigalloylglucose	19.53	635.0864	107 962 646 (2.16%)	3 546 009 (0.08%)
15	COC(=O)C1(CC(C(C(C1)OC(=O)C=CC2=CC(=C(C=C2)O)O)O)O)O	123483-19-2	Methyl chlorogenate	19.59	369.1172	2114 685 (0.04%)	13688 373 (0.30%)
16	C1=C(C=C(C(=C1O)O)O)C(=O)OCC2C(C(C(C(O2)OC(=O)C3=CC(=C(C(=C3)O)O)O)O)O)O)O	23363-08-8	1,6-bis-*O*-galloyl-β-D-glucose	19.70	483.0758	251 605 789 (5.04%)	8 383 446 (0.18%)
17	CC1C(C(C(C(O1)OCC2C(C(C(C(O2)OC3=C(OC4=CC(=CC(=C4C3=O)O)O)C5=CC(=C(C(=C5)O)O)O)O)O)O)O)O)O	41093-68-9	Myricetin-3-rutinoside	19.75	627.1544	98083 034 (1.90%)	17940 256 (0.39%)
18	CC(=O)OC1(CC(C2(C1C(OC=C2)OC3C(C(C(C(O3)CO)O)O)O)O)O)C	6926-14-3	8-*O*-Acetylharpagide	19.78	429.1359	12 707 623 (0.25%)	39216 825 (0.85%)
19	COC1=C(C=C2C(=C1O)C(=O)C=C(O2)C3=CC(=C(C=C3)O)O)OC4C(C(C(C(O4)C(=O)O)O)O)O)O	82657-12-3	β-D-Glucosiduronic acid, 2-(3,4-dihydro xyphenyl)-5-hydroxy-6-methoxy-4-oxo-4H-1-benzopyran-7-yl	20.16	395.0939	27823 579 (0.56%)	12271 382 (0.27%)
20	C1=CC(=C(C=C1C2=C(C(=O)C3=C(C=C(C=C3O2)O)O)O)OC4C(C(C(C(O4)CO)O)O)OC(=O)C5=CC(=C(C(=C5)O)O)O)O	53209-27-1	2'-*O*-galloylhyperin	20.17	617.1126	256 823 533 (5.14%)	11358 492 (0.25%)
21	C1=C(C=C(C(=C1O)O)O)C2=C(C(=O)C3=C(C=C(C=C3O2)O)O)OC4C(C(C(C(O4)CO)O)O)O	15648-86-9	Myricetin 3-galactoside	20.35	481.0964	143761 867 (2.88%)	10531 074 (0.23%)
22	C1C(C(C(C(O1)OCC2C(C(C(C(O2)OC3=C(OC4=CC(=CC(=C4C3=O)O)O)C5=CC(=C(C=C5)O)O)O)O)O)O)O)O	23284-18-6	Peltatoside (quercetin-3-*O*-arabino glucoside)	20.59	597.1439	252 993 403 (5.07%)	101001 688 (2.19%)

23	C1C(C(C(O1)OCC2C(C(C(C(O2OCCC3=CC=CC=C3)CO)O)O)O)(CO)O	371113-07-4	Sayaendoside	20.62	439.1569	294 250 466 (5.89%)	305140 167 (6.62%)
24	C(C1C(C(C(C(O1)OCC2C(C(C(O2)(CO)O)O)O)O)O)O)O	58166-27-1	Palatinose	20.84	325.0910	2161 600 (0.04%)	734 945 (0.02%)
25	C1=CC(=CC=C1/C=C/C(=C\2/C(=C(C(=O)C(C2=O)(C3C(C(C(C(O3)CO)O)O)O)O)(C4C(C(C(C(O4)CO)O)O)O)/O)O	78281-02-4	Hydroxysafflor yellow A	21.00	611.1595	423 917 474 (8.49%)	372294712 (8.07%)
26	C1=CC(=C(C=C1C2=C(C(=O)C3=C(C=C(C=C3O2)O)O)OC4C(C(C(C(O4)CO)O)O)O)O)O	482-36-0	Hyperoside	21.01	465.1015	569496 692 (11.41%)	230351 300 (5.00%)
27	C1C(C(C(C(O1)OCC2C(C(C(C(O2)OCCC3=CC=CC=C3)O)O)O)O)O)O	129932-48-5	β-D-Glucosyl-2-phenylethyl-6-*O*-β-D-xylopyranoside	21.14	439.1565	2 054 691 (0.04%)	11365 070 (0.25%)
28	CC1(C(C(C(C(O1)OCC2C(C(C(C(O2)OC3=C(OC4=CC(=CC(=C4C3=O)O)O)C5=CC(=C(C=C5)O)O)O)O)O)O)O)O	153-18-4	Rutin	21.15	611.1592	182 945 024 (3.66%)	211206 590 (4.58%)
29	CC(=O)NC(CC1=CNC2=CC=CC=C21)C(=O)O	87-32-1	*N*-acetyltryptophan	21.25	247.1071	1 860 401 (0.04%)	31686 457 (0.69%)
30	CC1=C(C(CCC1)(C)C)/C=C/C(=C/C(=O)O)/C	14398-42-6	(*E*,*E*)-3-Methyl-5-(2,6,6-trimethyl-1-cyclohexen-1-yl)-2,4-pentadienoic acid	21.44	443.1894	2 362 093 (0.05%)	44427 156 (0.96%)
31	C1=CC(=CC=C1C2=CC(=O)C3=C(O2)C=C(C(=C3O)C4C(C(C(C(O4)CO)O)O)O)O)O	38953-85-4	Isovitexin	21.47	433.1119	11963 439 (0.24%)	18199 468 (0.39%)
32	C1=CC(=C(C=C1C2=C(C(=O)C3=C(C=C(C=C3O2)O)O)O)O)O	117-39-5	Quercetin	21.62	303.0493	9263 420 (0.19%)	6 573 430 (0.14%)
33	C1C(OC2=CC(=CC(=C2C1=O)O)OC3C(C(C(C(O3)CO)O)O)O)C4=CC=C(C=C4)O	529-55-5	Naringenin-7-*O*-glucoside	21.63	435.1274	22 373 699 (0.45%)	138909 348 (3.01%)

34	C1=CC(=CC=C1C2=C(C(=O)C3=C(C=C(C=C3O2)O)O)O)OC4C(C(C(C(O4)CO)O)O)O	480-10-4	6"-*O*-L-arabinopyranosyl astragalin	21.77	581.1489	2 508 029 (0.05%)	40684 936 (0.88%)
35	CC1C(C(C(C(O1)OCC2C(C(C(C(O2)OC3=C(OC4=CC(=CC(=C4C3=O)O)O)C5=CC=C(C=C5)O)O)O)O)O)O)O	17650-84-9	kaempferol-3-*O*-rutinoside	21.78	595.1647	103 900 471 (2.08%)	118492 531 (2.57%)
36	C1=C(C=C(C(=C1O)O)O)C(=O)OCC2C(C(C(C(O2)OC(=O)C3=CC(=C(C(=C3)O)O)O)OC(=O)C4=CC(=C(C(=C4)O)O)O)OC(=O)C5=CC(=C(C(=C5)O)O)O)O	79886-50-3	1,2,3,6-tetragalloylglucose	21.86	787.0966	231 807 467 (4.64%)	4 140 064 (0.09%)
37	C1=CC(=C(C=C1C2=CC(=O)C3=C(C=C(C=C3O2)OC4C(C(C(C(O4)CO)O)O)O)O)OC5C(C(C(C(O5)CO)O)O)O)O	52187-80-1	Luteolin-7,3'-di-*O*-glucoside	22.12	609.1444	0 (0%)	193 827 (0.04%)
38	C1=CC=C(C=C1)C2=C(C(=C(C3=CC=CC=C3)C(=O)O)OC2=O)O	26548-70-9	Pulvinic acid	22.15	307.0447	5145 102 (0.10%)	34943 156 (0.76%)
39	C1=CC(=C(C=C1C2=CC(=O)C3=C(C=C(C=C3O2)OC4C(C(C(C(O4)CO)O)O)O)O)O)O	1268798	luteolin-7-*O*-β-D-glucoside	22.16	449.1066	263284 078 (5.27%)	213769 931 (4.64%)
40	CC1C(C(C(C(O1)OCC2C(C(C(C(O2)OC3=C(OC4=CC(=CC(=C4C3=O)O)O)C5=CC(=C(C=C5)O)OS(=O)(=O)O)O)O)O)O)O)O.[K+]	1486-70-0	3’-Methoxyquercetin-3-*O*-rutinoside	22.33	625.1745	2 807 934 (0.06%)	32042 186 (0.69%)
41	CC1C(C(C(C(O1)OC2C(C(C(O[C]2OC3=CC(=C4C(=C3)OC(=CC4=O)C5=CC(=C(C=C5)O)OC)O)CO)O)O)O)O)O	111133-90-5	7-*O*-neohesperidosyl Chrysoeriol	22.38	609.1804	0 (0%	196 837 (0.04%)
42	CC1=CC(=O)CC(C1(C=CC(C)OC2C(C(C(C(O2)CO)O)O)O)O)(C)C	54835-70-0	Roseoside	22.43	387.2001	6909 671 (0.14%)	18017 930 (0.39%)
43	C1=CC=C2C(=C1)C(=O)C3=C(C2=O)C(=CC(=C3)O)O	518-83-2	1,3-Dihydroxyanthraquinone	22.59	239.0190	25 438 392 (0.51%)	47110 390 (1.02%)

44	CC1(C=CC2=C(O1)C=CC3=C2OC(=CC3=O)C4=CC=CC=C4)C	64125-32-2	8,8-dimethyl-2-phenylpyrano[2,3-f]chromen-4-one	22.65	305.1125	272 436 (0.01%)	11677 199 (0.25%)
45	C1=C(C=C(C(=C1O)O)O)C(=O)OCC2C(C(C(C(O2)OC(=O)C3=CC(=C(C(=C3)O)O)O)OC(=O)C4=CC(=C(C(=C4)O)O)O)OC(=O)C5=CC(=C(C(=C5)O)O)O)OC(=O)C6=CC(=C(C(=C6)O)O)O	14937-32-7	β -Penta-*O*-galloyl-glucose	22.91	939.1073	53 919 155 (1.08%)	0 (0%)
46	COC1=C(C=CC(=C1)C=CC(=O)NCCCCN)O	501-13-3	Subaphyllin	23.01	314.1379	0 (0%)	870 665 (0.02%)
47	C1=CC(=CC=C1C=CC(=O)OCC2C(C(C(C(O2)OC3=C(OC4=CC(=CC(=C4C3=O)O)O)C5=CC=C(C=C5)O)O)O)O)O	20316-62-5	Tiliroside	23.04	593.1490	485 708 (0.01%	723 658 (0.02%)
48	C1=C(C=C(C(=C1O)O)O)C2=C(C(=O)C3=C(C=C(C=C3O2)O)O)OC4C(C(C(C(O4)COC(=O)C5=CC(=C(C(=C5)O)O)O)O)O)O	15648-86-9	Myricetin-3-*O*-β-D-galactoside 6''-*O*-gallate	23.18	631.0919	118 510 736 (2.37%)	8 806 699 (0.19%)
49	CCCCOC(=O)C(CC1=CC=CC=C1)N	15100-75-1	L-phenylalanine butyl ester	23.27	222.1483	332 365 866 (6.66%)	90019 584 (1.95%)
50	CC1=CC(=O)CC(C1CC(C)OC2C(C(C(C(O2)CO)O)O)O)(C)C	135820-80-3	Byzantionoside B	23.72	373.2196	13 907 102 (0.28%)	92036 025 (2.00%)
51	CC(C)C1(CCC(=CC1)CO)OC2C(C(C(C(O2)CO)O)O)O	16203-27-3	(2*R*,3*S*,4*S*,5*R*,6*S*)-2-(hydroxymethyl)-6-[4-(hydroxymethyl)-1-propan-2-ylcyclohex-3-en-1-yl]oxyoxane-3,4,5-triol	23.85	355.1718	268 591 559 (5.38%)	327376 033 (7.10%)
52	CC1C(C(C(C(O1)OCC2C(C(C(C(O2)OC3=CC(=C4C(=C3)OC(=CC4=O)C5=CC(=C(C=C5)OC)O)O)O)O)O)O)O)O	520-27-4	Diosmin	24.36	607.1649	0 (0%)	273 383 (0.01%)
53	CCC(C)C(=O)C(=O)NCCC1=CNC2=CC=CC=C21	183314-24-1	Nematophin	24.66	273.1648	3 474 799 (0.07%)	1 634 480 (0.04%)

54	C1=CC(=C(C=C1C2=CC(=O)C3=C(C=C(C=C3O2)O)O)O)O	491-70-3	Luteolin	24.68	287.0543	4595 397 (0.09%)	9 688 778 (0.21%)
55	CC(=O)OCC1C(C(C(C(O1)OC2C(C(C(OC2OC3=C(C4=C(C(=C3)O)C(=O)C=C(O4)C5=CC=C(C=C5)OC)O)CO)O)O)O)O)O	80680-48-4	4'-methoxyisoscutellarein7-[2”-O-(6”’-O-acetyl-β-D-allopyranosyl)-β-D-glucosyl]	25.03	667.1860	3 509 386 (0.07%)	178674 894 (3.88%)
56	C(CCCC(=O)O)CCCC(=O)OCC(CO)O	109421-77-4	9-(2,3-dihydroxypropoxy)-9-oxononanoic acid	25.22	261.1336	0 (0%)	582 638 (0.01%)
57	C1=CC(=CC=C1C2=CC(=O)C3=C(C=C(C=C3O2)OC4C(C(C(C(O4)CO)O)O)O)O)O	578-74-5	Apigenin-7-*O*-β-D-glucoside	25.36	431.0966	1 876 149 (0.04%)	1 578 292 (0.03%)
58	CC1C(C(C(C(O1)OC2=C(OC3=CC(=CC(=C3C2=O)O)O)C4=CC(=C(C(=C4)O)O)O)O)O)O	17912-87-7	Myricitrin	25.85	463.0863	324708 952 (6.50%)	108056 798 (2.34%)
59	CC(=CCC/C(=C/COC1C(C(C(C(O1)COC2C(C(C(O2)CO)O)O)O)O)O)O)/C)C	84534-32-7	Geranyl-6-*O*-α-L-arabinofuranosyl-*O*-β-D glucoside	25.96	471.2197	18 719 776 (0.37%)	83466 864 (1.81%)
60	CC(=CCC(C)(C=C)OC1C(C(C(C(O1)COC2C(C(C(CO2)O)O)O)O)O)O)C		(*R*)-Linalyl-β-vicianoside	26.20	471.2194	69 919 724 (1.40%)	250642 158 (5.44%)
61	C1C(OC2=CC(=CC(=C2C1=O)O)O)C3=CC=C(C=C3)O	67604-48-2	Naringenin	26.47	271.0604	61307 962 (1.23%)	114285 140 (2.48%)
62	OC1=C2C(C(C=C(C3=CC(O)=C(C=C3)O)O2)=O)=CC=C1O C4O(C(C(C(O4)O)O)O)CO	925701-05-9	8,3',4'-trihydroxyflavone-7-*O*-(6'-*O*-*p*-coumaroyl)-β-D-glucoside	26.89	593.1493	117 608 434 (2.36%)	216792 607 (4.70%)
63	CC1=C(C(CC(C1OC2C(C(C(C(O2)CO)O)O)O)O)(C)C)CCC(=O)C	17283-81-7	2-butanone, 4-[3-(β-D-glucopyranosyloxy)-4-hydroxy-2,6,6-trimethyl-1-cyclohexen-1-yl]	26.92	371.2043	4 451 363 (0.09%)	2 517 190 (0.05%)
64	CC(CCCCCCCCCC1CC2=C(C(=CC(=C2)O)O)C(=O)O1)O	154850-35-8	6,8-dihydroxy-3-(10-hydroxyundecyl)-3,4-dihydroisochromen-1-one	26.98	351.2133	38345199 (0.77%)	71191 586 (1.54%)

65	CC1C(C(C(C(O1)OCC2C(C(C(C(O2)OC3=C(OC4=CC(=CC(=C4C3=O)O)O)C5=CC(=C(C=C5)O)OC)O)O)O)O)O	604-80-8	Narcissin	27.23	623.1595	19122 741 (0.38%)	33449 197 (0.73%)
66	CCCCCCCCCCCC(=O)N(CCO)CCO	120-40-1	Lauryldiethanolamide | *N,N*-bis(2-hydroxyethyl)dodecanamide	27.88	288.2525	450 500 (0.01%)	49395 923 (1.07%)
67	CCCCCCCCCCCCCCC(C(C(CO)N)O)O	554-62-1	Phytosphingosine	28.69	318.2994	0 (0%)	450 011 (0.01%)
68	CC1C(C(C(C(O1)OC2=C(OC3=CC(=CC(=C3C2=O)O)O)C4=CC=C(C=C4)O)O)O)O	482-39-3	Afzelin	28.77	431.0968	23 368 330 (0.47%)	27836 625 (0.60%)
69	CC1=CC2=C(C(=C1)O)C(=O)C3=C(C2=O)C=C(C=C3O)O	518-82-1	Emodin	28.85	269.0447	10100 304 (0.20%)	7 322 516 (0.16%)
70	C1=CC=C(C=C1)C(=O)C=CC2C(C=C(C=C2O)O)O	1088-08-0	2',4',6'-Trihydroxydihydrochalcone	30.78	257.0811	204 060 (0.004%)	0 (0%)
71	C1=CC(=CC=C1C2=C(C(=O)C3=C(O2)C(=CC(=C3)O)O)O)O	3440-24-2	Tetrahydroxyflavone	31.12	285.0394	14 040 475 (0.28%)	183589 163 (3.98%)
72	CC1CCC(C=CC(=O)OC(CCC(C=CC(=O)O1)O)C)O	22248-41-5	Pyrenophorol	31.41	311.1673	2104 127 (0.04%)	2 137 819 (0.05%)
